# Studies of stability and characterization this enzyme bromelain in pineapple (Ananas comosus)

**DOI:** 10.1186/1753-6561-8-S4-P137

**Published:** 2014-10-01

**Authors:** Bianca Chaves Martins, Robson Rescolino, Diego de Freitas Coelho, Foued Salmen Espindola, Beatriz Zanchetta, Elias Basile Tambourgi, Edgar Silveira

**Affiliations:** 1 Universidade Federal de Uberlândia, Uberlândia, Brazil; 2 Universidade Estadual de Campinas, Campinas, Brazil

## Background

Bromelain is the generic name given to the set of derived endopeptidases belonging to members of the Bromeliaceae family, which belongs to the pineapple (Ananas comosus), being able to break the peptide bond, separating proteins and amino acids [1]. Bromelain possesses a wide range of therapeutic benefit as property of facilitating digestion of proteins, meat softening, ability to facilitate blood clotting [2] And economic importance related to the food industry and textiles and production of drugs resulting in an increase of its value [3]. Thus, the aim of this work is to evaluate the stability of the enzyme in relation to different temperatures and pH to make feasible the purification of the same.

## Methods

The enzyme extract was obtained from the peel, stem and leaves of (Ananas comosus). The plant tissue derived from the peel, stem and leaves of pineapple were processed extractor and then centrifuged at 10,000 g for 20 minutes at 4 ° C to remove insoluble material. For the assay of enzymatic activity was used azocasein method, wherein azocasein 1,0% (w / v) (Sigma) was solubilized in ethanol 4% (v / v) and 0.1 M phosphate buffer, pH 7.0, and used as a substrate. The assay mixture, containing 125 μL of substrate and 125 μL of enzymatic extract was incubated for 10 minutes at 37 ° C and the reaction stopped by adding 750 μL of trichloroacetic acid 5% (w / v). The samples were centrifuged at 4000g for 10 minutes and at a temperature of 5 ° C. The stability of bromelain was evaluated against various pHs (5.0, 6.0, 7.0, 8.0, and 9.0 and 10.0) and at different temperatures (5, 25, 35, 40, 45, 50, 55 and 60 ° C). And readout was taken in a spectrophotometer at 440 nm, and a unit of activity was defined as the amount of enzyme required to produce an increase in optical density by one unit at an interval of one hour.

## Results and conclusion

The enzyme activity increased with increasing temperature until it reaches 50 ° C, where it began to decline rapidly. Front pH changes, the activity increased to pH 7.0, where declined with the change of the same, raising and exhibiting a second peak of activity at pH 10.0. The optimum temperature and pH for activity was 50 ° C and pH 7.0, in which one observes the greatest activity of the enzyme bromelain. Thus, it was concluded that for this study the enzyme stability is interesting to work with these conditions.

## References

[B1] Souza OP Coutinho AC Torres TLR Avaliação econômica da produção do acabaxi irrigado cv Smooth Cayenne no Cerrado, em Uberaba-MG Revista Universo Rural 2010 30 1 00 00

[B2] Abílio GMF Holschuh HJ Bora PS Oliveira EFD Extração, atividade da bromelina e análise de alguns parâmetros químicos em cultivares de abacaxi Revista Brasileira de Fruticultura 2009 31 1117 1121 10.1590/S0100-29452009000400027

[B3] Coelho D Barbosa SA Silveira E Souza RR Tambourgi E Biosurfactant Production from Unconventional Resources: a Short Overview International Review of Chemical Engineering 2012 4 2 175 183

